# Genes Encoding Enzymes Involved in Ethanol Metabolism

**DOI:** 10.35946/arcr.v34.3.09

**Published:** 2012

**Authors:** Thomas D. Hurley, Howard J. Edenberg

**Affiliations:** **Thomas D. Hurley, Ph.D.,***is a Chancellor’s Professor in the Department of Biochemistry and Molecular Biology, Indiana University School of Medicine, Indianapolis, Indiana.*; **Howard J. Edenberg, Ph.D.,***is a Distinguished Professor and Chancellor’s Professor in the Department of Biochemistry and Molecular Biology and the Department of Medical and Molecular Genetics, Indiana University School of Medicine, Indianapolis, Indiana.*

**Keywords:** Alcohol consumption, alcohol dependence, alcoholism, ethanol metabolism, genetic factors, protective factors, risk factors, DNA, genetics, genetic variance, enzymes, acetaldehyde, alcohol dehydrogenase (ADH), aldehyde dehydrogenase (ALDH), single nucleotide polymorphisms (SNPs), esophageal cancer

## Abstract

The effects of beverage alcohol (ethanol) on the body are determined largely by the rate at which it and its main breakdown product, acetaldehyde, are metabolized after consumption. The main metabolic pathway for ethanol involves the enzymes alcohol dehydrogenase (ADH) and aldehyde dehydrogenase (ALDH). Seven different ADHs and three different ALDHs that metabolize ethanol have been identified. The genes encoding these enzymes exist in different variants (i.e., alleles), many of which differ by a single DNA building block (i.e., single nucleotide polymorphisms [SNPs]). Some of these SNPs result in enzymes with altered kinetic properties. For example, certain ADH1B and ADH1C variants that are commonly found in East Asian populations lead to more rapid ethanol breakdown and acetaldehyde accumulation in the body. Because acetaldehyde has harmful effects on the body, people carrying these alleles are less likely to drink and have a lower risk of alcohol dependence. Likewise, an ALDH2 variant with reduced activity results in acetaldehyde buildup and also has a protective effect against alcoholism. In addition to affecting drinking behaviors and risk for alcoholism, ADH and ALDH alleles impact the risk for esophageal cancer.

The duration and extent of the body’s exposure to beverage alcohol (i.e., ethanol) is the primary determinant of ethanol’s pleiotropic effects on human health (Edenberg 2007). The time course of its concentration and the concentration of its byproducts in the tissues and the circulation, and, consequently, its effects, are determined mainly by the rate of ethanol’s processing (i.e., metabolism) in the body. Ethanol can be metabolized in several reactions, but this review focuses on the primary pathway through which it is eliminated from the systemic circulation. In humans, this primary pathway of ethanol metabolism involves oxidation to acetaldehyde by the enzyme alcohol dehydrogenase (ADH). The acetaldehyde then is further oxidized by the enzyme aldehyde dehydrogenase (ALDH) to acetate, which is either excreted in the urine or reincorporated into intermediary metabolism as acetyl-CoA. The hydrogen atoms that are released during these reactions are used to generate a compound called reduced nicotinamide dinucleotide (NADH), with two NADH molecules produced per molecule of acetate generated. The resulting NADH and acetate are thought to provide both the excess reducing equivalents and excess acetyl-CoA that are needed as starting material for fatty acid synthesis, which results in the development of fatty liver disease if high amounts of alcohol are ingested over time.

Both ADH and ALDH exist in different variants with different levels of activity, therefore resulting in different rates of ethanol metabolism. This article discusses how these differences influence a person’s sensitivity to ethanol’s effects and his or her risk of alcohol dependence.

## ADH Variants

Humans have seven ADHs that can carry out the first step in alcohol metabolism. The genes encoding these enzymes all are localized on chromosome 4 in a head-to-tail array about 370 kb long. The enzymes produced from these genes all differ slightly in their activities (see table 1):

- The *ADH1A*, *ADH1B*, and *ADH1C* genes^1^ produce closely related proteins that function as homo- and heterodimers (Hurley et al. 2002); their kinetic properties, tissue localization, and developmental expression all support major roles in oxidative ethanol metabolism in the liver.
- The *ADH4* gene is expressed almost exclusively in the liver (Hurley et al. 2002), where it contributes significantly to ethanol oxidation at higher levels of consumption.
- The product of the ubiquitously expressed *ADH5* gene is the glutathione-dependent formaldehyde dehydrogenase (also known as nitrosoglutathione reductase [GSNOR]). The physiological substrates for ADH5 (α-ADH) are compounds (i.e., adducts) formed during the reaction between glutathione and formaldehyde and between glutathione and nitric oxide (Que et al. 2005; Sanghani et al. 2000). The main functions of this enzyme are to oxidize formaldehyde to formic acid and to terminate nitric oxide signaling. The human ADH5 enzyme is nonsaturable with ethanol as a substrate, unless medium-chain fatty acids are present in the assay (Engeland et al. 1993), and was originally thought to contribute little to ethanol oxidation. However, its relatively high maximal velocity, coupled with its ubiquitous expression pattern and the high concentrations of ethanol found in gastric tissues, has led some researchers to suggest that it plays a significant role in first-pass metabolism (Lee et al. 2003).
- Although the *ADH6* gene has been identified, there are as yet no physiological data on the functions of the ADH6 enzyme.
- The *ADH7* gene has a limited expression pattern and mainly is found in endothelial cells, such as those lining the esophageal and stomach tissues, as well as during embryonic development when it may contribute to the metabolism of retinol, a form of vitamin A (Hurley et al. 2002). In adults, ADH7 has been implicated in the first-pass metabolism of ethanol taking place in the gastroesophageal tissues, before the ethanol is delivered to the liver via the portal vein (Hurley et al. 2002).

The *ADH* gene cluster contains many single-nucleotide polymorphisms (SNPs)—that is, sites in which the DNA sequence differs by a single building block (i.e., nucleotide) from the reference sequence. Some of these variations result in an altered amino acid sequence of the encoded enzyme and therefore are considered functional or coding SNPs (cSNPs). Detailed functional studies are lacking for all these cSNPs except those that give rise to the *ADH1B* and *ADH1C* gene variants (i.e., alleles).

### The ADH1B Alleles

The three most studied alleles of *ADH1B* usually are referred to as ADH1B*1 (the reference allele, which encodes the β_2_ form of the enzyme and carries the amino acid arginine [Arg] at positions 48 and 370 in the amino acid chain), ADH1B*2 (encoding β_2_ and carrying histidine [His] at position 48: ADH1B-His48Arg370; rs1229984), and ADH1B*3 (encoding β_3_ and carrying cysteine [Cys] at position 370: ADH1B-Arg48Cys370; rs2066702). The encoded enzyme variants differ significantly in the ethanol concentrations they require for maximal function and in how fast they metabolize the ethanol (i.e., in their kinetic properties), with both ADH1B*2 and ADH1B*3 encoding enzymes with faster turnover (i.e., higher *V*_max_) than the reference allele (see table 1). One study of Japanese alcoholics who checked into a hospital 1 day after heavy drinking showed that those who carried two copies of (i.e., were homozygous for) the ADH1B*1 allele still had significant blood ethanol concentrations (BECs), whereas those who carried at least one ADH1B*2 allele had very low BECs, consistent with a more rapid ethanol metabolism by ADH1B*2 (Yokoyama et al. 2007). The current model posits that more rapid oxidation of ethanol at least transiently elevates acetaldehyde levels in one or more tissues. Because acetaldehyde has toxic or at least unpleasant effects on the body, leading to a flushing response after alcohol consumption, this acetaldehyde accumulation is thought to produce aversion that tends to limit heavy alcohol consumption by people who carry at least one ADH1B*2 allele.

The ADH1B*2 allele is very common in East Asian population, where it is the major allele with a frequency of 75 percent among Japanese and Chinese individuals (Eng et al. 2007; Li et al. 2007). It also is relatively common in the Middle East (frequency 20 percent) but is uncommon elsewhere in Europe or Africa. In general, its allele frequency is less than 4 percent in populations of European descent, and it was not found in any of the 90 individuals of European descent that were studied in a large genotyping project (i.e., HapMap). Likewise, it is absent from most African populations. As a result, the allele is not included in most of the genotyping arrays used in genome-wide association studies (GWASs).

In the Asian populations where ADH1B*2 is common, there is very strong evidence that it is protective against alcohol dependence (Chen et al. 1999; Edenberg 2007; Li et al. 2007; Thomasson et al. 1991; Whitfield 2002). An analysis among Han Chinese in Taiwan showed that the relative risk of alcohol dependence was reduced to 0.2 if a person carried a single ADH1B*2 allele and to 0.12 for homozygotes (Chen et al. 1999). A more recent meta-analysis similarly showed a very strong protective effect for the ADH1B*2 allele in Asians (odds ratio ∼ 0.44; *P* < 10^−36^) (Li et al. 2007). It has been more difficult to detect the effect of ADH1B*2 in Europeans; the meta-analysis by Li and colleagues (2007) showed an odds ratio of 0.65 and *P* = 0.04 in alcohol-dependent people without secondary disease. A study in European Americans found that each ADH1B*2 allele lowered the number of symptoms of alcoholism as specified in the *Diagnostic and Statistical Manual of Mental Disorders, Fourth Edition* (DSM–IV) and also reduced the maximum number of drinks consumed in one sitting (Sherva et al. 2009). Another analysis across three large, well-characterized samples of European Americans demonstrated a strong effect of ADH1B*2, close to what is seen in Asian populations (odds ratio <0.4; *P* < 10^−8^) (Bierut et al. 2012). The ADH1B*2 allele also was associated with hypersensitivity to alcohol in a Scandinavian population (Linneberg et al. 2010), as well as with significantly less drinking before and during pregnancy in European women (Zuccolo et al. 2009). Finally, both maternal and fetal ADH1B*2 reduced the risk for fetal alcohol spectrum disorders in a mixed population from South Africa (Warren and Li 2005).

The ADH1B*3 allele is relatively common in Eastern African populations, with frequencies of 27 percent among Yoruba, 14 percent among Luhya, and 24 percent among people of African ancestry in the Southwestern United States but only 4 percent among Maasai in Kenya. In contrast, the allele is rare elsewhere and is not found in the European or Asian HapMap samples. Fewer studies of alcohol dependence have been conducted among people of African ancestry, but ADH1B*3 has been shown to be protective against alcohol dependence (Edenberg 2007; Edenberg et al. 2010). Moreover, the presence of ADH1B*3 in pregnant women leads to less drinking at conception and fewer adverse effects in the children born to these women (Jacobson et al. 2006).

### The ADH1C Alleles

*ADH1C* also has cSNPs, of which ADH1C*1 and ADH1C*2 are the most studied. These two alleles differ at two sites, resulting in two amino acid changes: the enzyme encoded by ADH1C*1 (γ1-ADH) has Arg at position 272 and isoleucin (Ile) at position 350, whereas that encoded by ADH1C*2 (γ2-ADH) has glutamine (Gln) at position 272 and valine (Val) at position 350 (Osier et al. 2002).^2^ The kinetic differences between γ1-ADH and γ2-ADH are smaller than those between the ADH1B isozymes (table 1). Studies in Asian populations have shown an association between *ADH1C* alleles and alcoholism, but the protective effect of ADH1C*1 in that population may be explained in large part by its coinheritance with the ADH1B*2 allele. A SNP in the *ADH1C* gene that is always inherited together with (i.e., is in complete linkage disequilibrium) with one of the amino acid changes at this locus was associated with alcoholism in two GWASs candidate gene substudies on people of European descent, but the difference did not reach statistical significance for genome-wide analyses (Kendler et al. 2011; Treutlein et al. 2009). The ADH1C*1 allele has been associated with increased risk for alcohol-related cancer, particularly in people who consume alcohol (Seitz and Meier 2007).

Many other variations in and around the seven *ADH* genes have been associated with risk for alcohol dependence or alcohol-related traits. Among these, variations in and near *ADH4* are among the most widely replicated associations with alcohol dependence, in several populations. Moreover, noncoding SNPs in the region of *ADH1A*, *ADH1B*, and *ADH1C* have been associated with alcoholism and drinking phenotypes, as have SNPs in *ADH7* and *ADH5*. Some non-coding SNPs have been shown to affect gene expression in cultured cells and tissues (i.e., in vitro) (Chen et al 2005; Pochareddy and Edenberg 2010, 2011), and it is likely that many different variations in this region also affect the level of expression of the different ADH enzymes in the intact organism (i.e., in vivo), thereby influencing ethanol metabolism (in some cases possibly only in specific tissues), its physiological effects, and, ultimately, drinking behavior and risk for alcoholism. Without molecular studies, however, detailed analyses of which SNPs might be functional are difficult because many of the *ADH* variations are inherited together with nearby SNPs as haplotypes.

## ALDH Variants

The acetaldehyde produced by the action of one or more ADH enzymes must be oxidized efficiently by one or more ALDH enzymes in order for the cell/tissue to maintain non-toxic levels of this reactive molecule. Even transient elevation of acetaldehyde can provoke aversive reactions in people whose ALDH activity is reduced either genetically or pharmacologically. Unlike the human *ADH* genes, the *ALDH* genes are not localized to a single chromosome. Humans have 18 genes encoding for members of the ALDH enzyme superfamily (Jackson et al. 2011). Three of these—*ALDH1A1*, *ALDH1B1*, and *ALDH2*—are most relevant to acetaldehyde oxidation (table 2). The three ALDH enzymes encoded by these genes share more than 68 percent amino acid sequence identity; all three enzymes function in the cell as homotetramers. The ALDH1A1 enzyme is found in the cytosol, whereas both ALDH1B1 and ALDH2 are produced in the nucleus but have leader sequences that direct them to cell components called mitochondria, where they exert their functions in the mitochondrial interior (i.e., the matrix) (Jackson et al. 2011). Of the three isoenzymes, ALDH2 seems to carry out most of the oxidation of ethanol-derived acetaldehyde, as demonstrated by the effects of its inhibition by activated forms of the medication disulfiram (Antabuse^®^) and by the effects of a functional polymorphism commonly found in East Asian populations (ALDH2*2)*,* in which a critical glutamate is substituted by a lysine residue at position 504 of the precursor protein (487 of the mature protein) (ALDH2-Lys504; rs671) (Hurley et al. 2002). With both disulfiram and the ALDH2*2 enzyme, ALDH2 activity is severely compromised, resulting in increased levels of acetaldehyde, which enters the systemic circulation and initiates the commonly observed facial flushing syndrome. In vitro kinetic analyses also are consistent with the key role of ALDH2, demonstrating that the ALDH2 isoenzyme has the highest catalytic efficiency for acetaldehyde oxidation (Hurley et al. 2002). The ALDH2*2 allele is relatively common in East Asia (frequencies of 12 to 41 percent [Li et al. 2009]), where it has a very strong effect on risk for alcoholism. Thus, people who carry one copy of the inactive allele are strongly protected against alcoholism (odds ratio from 0.5 to 0.12 [Chen et al. 1999; Thomasson et al. 1991]), and homozygotes are almost completely protected.

It is likely that the ALDH1A1 and ALDH1B1 enzymes significantly contribute to acetaldehyde metabolism only in situations where ALDH2 is inactivated, either pharmacologically or because of the presence of the ALDH2*2 allele. The *K*_M_ values^3^ of the ALDH1A1 and ALDH1B1 enzymes exceed those of the ALDH2 enzyme by at least 100-fold, and, thus, are not likely to be operating at full capacity when acetaldehyde levels are kept at the usual physiological state (i.e., below 5 μmol/L) (Klyosov et al. 1996; Stagos et al. 2010). Numerous polymorphisms have been identified for the *ALDH1A1* gene, and linkage to alcohol-related phenotypes has been found in both European populations (rs8187974 [Lind et al. 2008; Sherva et al. 2009]) and people of Indo-Trinidadian background (ALDH1A1*2, rs67952887 [Moore et al. 2007]). In addition, a functional polymorphism (i.e., rs2228093) in the *ALDH1B1* gene found in northern European populations seems to correlate with alcohol-aversive reactions (Husemoen et al. 2008; Linneberg et al. 2010), suggesting that the ALDH1A1 and ALDH1B1 isoenzymes contribute to ethanol metabolism under typical ethanol loads even in populations where the ALDH2*2 allele virtually is nonexistent.

## Cytochrome P450 Isoenzymes

Although the ALD/ALDH system is the primary pathway of ethanol metabolism in the body, another system called the microsomal ethanol oxidizing system (MEOS) also contributes to ethanol metabolism, particularly in alcoholics in whom chronic ethanol exposure induces higher expression levels of the enzymes involved. The primary component of the MEOS is cytochrome P450 2E1 (CYP2E1). Increased production of acetaldehyde through this pathway is associated with increased risk for liver damage (Lu and Cederbaum 2008), presumably because of the propensity of the P450 enzymes to produce reactive oxygen species as a byproduct of the catalytic activation of molecular oxygen. Until recently, most studies did not find a significant correlation between CYP2E1 polymorphisms and alcohol elimination rates or alcohol-induced liver injury. Recent work in India, however, has found a significant association between the CYP2E1*B5 allele (rs2031920) and alcoholic liver cirrhosis (Khan et al. 2009, 2010). These studies raise the possibility that additional associations exist between *CYP2E1* polymorphisms and alcohol-induced liver disease, warranting more detailed study in other populations.

## Alcohol Metabolism and Cancer

In addition to affecting drinking behaviors and risk for alcoholism, *ADH* and *ALDH* alleles affect the risk for esophageal cancer. In a meta-analysis of studies (primarily of East Asian populations), the presence of ADH1B*1 was associated with a higher risk for esophageal cancer even in men who drank little or rarely and had a greater effect in heavier drinkers (Yang et al. 2010). Moreover, although the presence of ALDH2*2 alleles reduces drinking, the risk for esophageal cancer is elevated among those who drink despite carrying these alleles. Finally, a large study of European subjects showed that ADH1B*2 and an allele in *ADH7* (the minor allele of rs1573496) independently were protective against upper aerodigestive cancers, particularly among heavier drinkers (Hashibe et al. 2008).

## Conclusions

The onset of the genomics era has initiated a rapid increase in researchers’ ability to find and analyze polymorphisms within the enzymes responsible for ethanol metabolism. In fact, the rate of discovery of polymorphisms in and near these genes far outpaces the ability to functionally characterize them. Future studies of the expression and kinetic properties of the variant enzymes are important. In particular, methodologies for rapidly and accurately determining protein expression levels of specific forms are needed. For example, a commonly used approach to identify individual problems—that is, the use of specific antibodies—has not yet worked with the ADH1A, ADH1B, and ADH1C proteins because these proteins are highly conserved, with their sequence at the protein level more than 93 percent identical; moreover, many of the sequence changes are located within functional sites and not optimally situated for antibody recognition. Knowledge of expression level changes, however, will be critical to develop models for predicting the metabolic consequences of both the currently characterized functional polymorphisms and those that are yet to be discovered.

## Figures and Tables

**Table 1:** Kinetic Constants for Ethanol Oxidation by Human Alcohol Dehydrogenases^1^

**Gene (Enzyme)**	**K_M_ (mM)**	**V_max_ (min^−1^)**	**% liver contribution^2^ at 22 mM ethanol^3^**
ADH1A (αα)	4	20	8.1
ADH1B*1 (ADH1B-Arg48Arg370; β1β1)	0.05	4	21.8
ADH1B*2 (ADH1B-His48Arg370; β2β2)	0.9	350	−2
ADH1B*3 (ADH1B-Arg48Cys370; β3β3)	40	300	−2
ADH1C*1 (ADH1C-Arg272, Ile350; γ1γ1)	1	90	41.5
ADH1C*2 (ADH1C-Gln272, Val350; γ2γ2)	0.6	40	−2
ADH4 (ππ)	30	20	28.6
ADH5 (χχ)	>1,000	100	<1
ADH7 (σσ)	30	1800	<1

1 Data from Hurley, Edenberg and Li, 2002.

2 Calculated for an individual homozygous for both ADH1B*1 and ADH1C*1; expression data for polymorphic ADH variants are uncertain.

3 22.7 mM corresponds to a blood alcohol concentration of 100 mg/dL

**Table 2:** Kinetic Constants for Acetaldehyde Oxidation by Human Aldehyde Dehydrogenases

**Enzyme**	**K_M_ (μM)**	**V_max_ (min^−1^)**	**V_max_ (min^−1^ μM^−1^)**
ALDH1A1	180	380	2.1
ALDH1B1	55	40	0.7
ALDH2*1	0.2	280	1400
ALDH2*2	1.4	20	14

1 Data for ALDH1A1 and ALDH2*1 from Klyosov, 1996; data for ALDH2*2 oxidation of propionaldehyde from Farrés et al., 1994 and data for ALDH1B1 from Stagos et al., 2010.

## Footnotes

1 By convention, the names of genes are written in italics, whereas the names of the corresponding proteins are written in normal font.

2 Another allele, *ADH1C*3,* which carries threonine instead of proline at position 352 on the background of *ADH1C*2* has been reported in Native Americans (Osier et al. 2002).

3 K_M_ is the concentration of an enzyme’s substrate—in this case ethanol—at which the rate of the enzyme reaction is half the maximum rate. The lower the K_M_, the more efficient the enzyme is toward that substrate.
